# Two Peculiar Cases of Hernia1Read before the Medical Society of the County of Erie, January 8, 1895.

**Published:** 1895-02

**Authors:** Clayton M. Daniels

**Affiliations:** 315 Jersey Street; Buffalo, N. Y.


### TWO PECULIAR CASES OF HERNIA.[1]

By CLAYTON M. DANIELS, M. D., Buffalo, N. Y.

In this brief paper it is not my intention to go into a dissertation upon hernia, interesting as the subject is in all its bearings, but to put upon record two cases which are novel if not unique.

Case I. On May 23, 1893, C. Munjella, 42 years old, married, an Italian laborer, while holding a "scraper" drawn by horses in the erection of a railway earthwork, was forcibly struck in the left groin by one of the handles. The blow caused great pain at the time, and a prominent swelling at once appeared. He continued work for two or three hours until suffering forced him to go home, where he remained for three days without attention, except the application of some household remedies. At the end of the third day he came to my office, walking with difficulty and well bowed over at every step.

Upon examination I immediately noted the absence of testicles in the scrotum, and the patient stated that he "never had any," so far as he knew, although he was a married man and sexually strong, but without children.

---

1. Read before the Medical Society of the County of Erie, January 8, 1895.

Attention being turned to the swelling in the groin, it was not hard to diagnosticate a full-sized testicle lodged in the inguinal canal. So long a time had elapsed since the injury that the swelling and inflammatory conditions present had occluded the internal ring so that a reduction into the abdominal cavity was not possible, and the cord was too short to allow of its progress farther toward the scrotum.

I directed him to the hospital, where later in the day I removed the testicle, leaving the stump of the cord carefully adjusted with a stitch so as to practically plug the internal ring from the inner side, making a radical operation so far as preventing the escape of any abdominal viscera hereafter. He promptly recovered and returned to duty in twenty days. He has since reported that he is perfectly well, and that for all practical purposes his sexual abilities are unimpaired.

It seems a peculiar coincidence that a man with non-descent of the testes should receive an injury necessitating partial castration.

On December 10, 1894, I was invited by your president to go to East Aurora prepared to operate for hernia. I found the patient to be a lady, Mrs. H., with the following history:

Case II. Age 37, married at 16 years, has had eight children, the two last being twins. Had an inguinal hernia upon the right side for thirteen years, but which caused but little trouble, as a truss had been worn satisfactorily.

On December 5th last, she reported to Dr. Gail that the hernia had been down for three weeks and a new truss would be required. She came to this city and consulted a well-known dealer in those articles and who tried to reduce the hernia, but failed. She secured the truss, went home and consulted Dr. Gail, complaining of pain in the back in addition to that in the groin, which had become so very tender that no amount of pressure could be tolerated, which made a critical examination difficult. A tumor the size of a hen's egg presented, which showed evidence of fluid, surrounding a firm lump which refused to be returned into the abdominal cavity. Bowels had been fairly regular and no nausea; these conditions were present up to the time I first saw her, although she was getting restless, not sleeping well, bowels somewhat tympanitic, pulse 80, temperature normal.

I felt certain that we were to meet with something out of the usual line of hernia, and think that opinion was shared by Dr. Gail.

Operation in the usual way, going down and opening the sac freely, from which escaped about two ounces of serum, and at the bot tom of the sac and very adherent nestled an ovary which had tried to escape via the inguinal canal. The internal ring had become closed by inflammatory adhesions, and after a little careful dissection I had the satisfaction of having performed ovariotomy without opening the abdominal cavity. I closed the ring with the ovarian ligament, which was quite large, in the same manner as I did with the spermatic cord in the first case described, anticipating a radical cure of the intestinal hernia previously occupying the canal.

Reports upon the case show that the wound closed by primary union, and that the patient has made a rapid recovery.

315 Jersey Street.

